# Report of Clinical Observations on Continued (Typhus and Typhoid) Fever

**Published:** 1850-08

**Authors:** Austin Flint

**Affiliations:** Prof. of the Principles and Practice of Medicine, and Clinical Medicine, in the University of Buffalo


### BUFFALO MEDICAL JOURNAL

AND

### MONTHLY REVIEW.

VOL. 6. AUGUST, 1850. NO. 3.

### ORIGINAL COMMUNICATIONS.

ART. I.—*Report of Clinical Observations on Continued (Typhus and Typhoid) Fever. Based on an Analysis of Fifty-Two Cases.* By AUSTIN FLINT, M. D., Prof. of the Principles and Practice of Medicine, and Clinical Medicine, in the University of Buffalo.

Having preserved histories of a considerable number of cases of Continued Fever, I propose to analyze them, and report the results in a series of articles for this Journal.

The first step in the analytical investigation, is to separate and classify all the important particulars embraced in the histories of the cases that have been collected. This has been already done. All the important circumstances occurring in the progress of every case (so far as recorded) have been selected and distributed in appropriate divisions. The labor which even this has required, can be best appreciated by those who are practically conversant with such tasks. In order to collocate the facts in the classes to which they belong, it was necessary that the examination of the history of each case should be repeated from twenty to thirty times. Multiplying the whole number of cases by the number of perusals of each case, will afford some idea of the tediousness of the process.

This is but a preliminary stage of the analysis. To develop useful results, the details thus brought into juxtaposition must be farther investigated. How shall this be done? in other words, how are these classified facts to be interrogated? Some may possess interest and value in themselves—isolated from the others—but, for the most part, it is by *enumeration* and *comparison* that the fruits of the analysis are to be acquired. It will be an object to determine, whenever practicable, the numerical proportions in which the different traits, symptoms, etc., belonging to the general history of the disease, respectively occur. That is to say, with regard to each of these particulars, in how many of the whole number of cases analyzed is it found to be present. This result is obtained by the simple process of counting. In this way is ascertained the numerical ratio which each bears to the whole number of cases, and, by comparison with each other, the mutual relations of all of them in this respect. By such enumerations, sufficiently often repeated, are developed the laws of a disease in so far as concerns the relative frequency of the production of the various particulars which compose its natural history. And the knowledge of these laws has other advantages than merely the gratification of a taste for statistics. If certain events, or symptoms, be proved to be to be constantly associated with a disease, and the same events, or symptoms, be proved not to be present, with any degree of constancy, in other diseases, it is a logical inference, that some very intimate connection subsists between the events, or symptoms, and the disease. This is an important result of numerical analysis, notwithstanding we are not able to penetrate farther by this method of investigation, and explain the nature of the bonds which bind these events, or symptoms, to the disease. The knowledge of the existence of such connections is certainly important as a point of departure for other investigations instituted with a hope of penetrating deeper into the mysteries of pathology. In like manner, events which are proved by enumeration to be not constant, but more or less frequent in their occurrence, may be considered as proving contingent relations to the disease in proportion to their frequency, or they may be proved to be purely accidental; and thus may be determined, not only what particulars intrinsically belong to the disease, but the relative degrees of relationship which other particulars sustain to it, and that certain events are to be excluded as having no real connection with the disease whatever. This kind of knowledge, moreover, has an important bearing on diagnosis, for, in proportion as an event or symptom is constant, or more or less frequent, is its diagnostic value to be estimated, provided, always, that it does not occur, or is far less frequent in its occurrence, in other diseases. Numerical analysis, therefore, tends to establish the relative importance of the several symptoms of disease as diagnostic criteria of the presence of that disease.

To follow out the trains of inquiry to which the foregoing considerations lead, would be to engage in discussions respecting the resources and limitations of the "*numerical system*," foreign to my present purpose.

Another mode of interrogating the facts contained in a collection of recorded cases of disease, is to compare the different cases with each other as respects associated symptoms. We find, for example, in a greater or less number of cases, a certain symptom; now, in the cases which present this symptom, do we always find certain other symptoms associated, and in the cases not presenting the symptom, are the associated symptoms absent? If analytical examination establishes any such relations, they involve, as a logical inference, some dependent connection between the symptoms thus associated in different cases, although the problem still remains to be solved, which of the symptoms are causes, and which effects, or whether all may not be alike effects of an unobserved, or inappreciable cause.

Again, the cases may be divided into several groups, and comparisons instituted between the groups, in order to ascertain what phenomena, or facts, belong to one or more groups, which do not belong to the others. We may thus be led to establish relations between the distinctive features upon which the division into groups was based, and other circumstances less obvious to observation, which the analysis and comparison reveal. Thus, we may separate fatal cases from those not fatal, and if we should chance to find in all the fatal cases certain characters which are not present in those cases which are not fatal, it would be a fair presumption, to say the least, that some connection existed between these characters and the fatal issue; at all events, knowledge of these characters would have an important practical bearing on the prognosis.

These few remarks are offered, by way of introduction, simply to suggest some of the leading objects to be kept in view in an analytical study of the data embraced in the records of clinical observations, foregoing discussions respecting the nature and scope of the logical principles involved in the application of what is known as the *numerical system* to investigations of disease. The latter is an highly interesting and important subject, but it is altogether too profound and extensive to be treated in an incidental connection. The value of this method of investigating medical truth is doubtless as much exaggerated by some, as it is underrated by many. In general terms, its advantages may be said to consist, no only in the development of a much greater number of results, but in rendering medical observation and experience vastly more precise and accurate. But it is hardly less desirable to understand its limitations, than to appreciate its advantages; and, in general terms, it furnishes data for delineating with completeness and correctness the *natural history* of diseases, it discloses relations between the events, or symptoms, of disease, it leads to a knowledge of laws regulating the succession of morbid phenomena; but it fails to penetrate the hidden springs upon which the observable characters of disease are dependent, it cannot unfold the links connecting those phenomena which are found to sustain relations with each other, and it leaves unexplained the laws the existence of which it declares.

To be satisfied of the very great utility and importance of recording cases, and subjecting them to analytical examination, it is only necessary to compare the general impressions which are formed while cases are under observation, with the results obtained, subsequently, by enumeration and comparison. These results are frequently as unexpected to the analyst, as to others: they often conflict directly with the anticipations derived from observing and recording the cases while in progress. This fact points to the great source of error in the medical experience of those whose inductions are based on facts retained in the memory alone. It is exceedingly difficult, as the phenomena of disease pass under observation, for the mind to resist becoming committed to generalizations which are often immature and erroneous; and when the mind is thus committed, it is equally difficult to continue observations fairly and impartially. Every person who studies the operations of mind in others, or who candidly reviews his own intellectual experience, must be convinced of the truth of this remark. How common is it to hear assertions made in the most positive manner, that in all cases of a particular disease, certain things are so and so—assertions made to sustain some peculiar views of pathology, diagnosis, or therapeutics; when a few observations by one who is unbiassed, or skeptical as to the correctness of the assertions, suffice for their disapproval. It does by no means follow that such rash declarations denote either dishonesty or stupidity; but they show that the mind, without vigilant watching, imposes upon itself. The prime error is in arriving at conclusions too hastily, without sufficient data, or proper investigation. So soon as this false step is taken, the mind is constantly seeking for evidence to sustain its position. Facts incompatible with such a position are not only overlooked, or excluded from the memory, but, sometimes, by an obliquity, and almost perverseness of judgment, even appear to sustain the false views which are pertinaciously cherished. This, it will be admitted, is a highly colored representation, but it is not a fancy sketch; exemplars are not so rare as not to be occasionally met with; of a lesser degree of the self-deception described, illustrations are abundant, and there are few so fortunate as to escape altogether the influences, alike imperceptible and untoward, of erroneous preconceptions, upon the powers both of observation and ratiocination. Hence originate the "false facts" with which medicine abounds, and that *experience* which is worse than valueless. The practice of recording observations, and submitting them to careful analysis, is of inestimable value, not only in the greater number, and better character of the results developed, but in cultivating a habit of mind which precludes error by withholding judgments, and accepting only as truth results elicited by adequate, trustworthy investigations. The whole philosophy of this subject is admirably embodied in the sentiment of Rousseau, selected by Louis as the motto for his work on Fever:—"*Je sais que la verite est dans les choses, et non dans mon esprit que les juge, et gue moins je mets du mien dans les jugements que j'en porte, plus je suis sur d'approcher de la verite.*"

After the incomparable work of Louis giving the results of his researches on Continued Fever compared with other acute diseases, and subsequent reports by several distinguished observers, it may perhaps appear a gratuitous, if not a presumptuous undertaking to make choice of cases of this affection, as a subject for analytical study. The elucidation, however, which the disease has already received, renders it more interesting, and scarcely less important, to prosecute continued researches. Were the results to be the same as those previously obtained, the labor would by no means be uselessly bestowed, since to confirm truth, is often very desirable, although a humbler attainment than the discovery of it. But other advantages are secured by a series of analyses of cases of the same affection occurring at different periods and places, and under a variety of circumstances. If the results are not uniform, the variations are to be attributed to the disturbing or modifying influences incident to time, situation, and other circumstances; and we may thus learn what belongs to the disease *per se*, and what is due to extrinsic causes. It is at once obvious that this kind of knowledge is highly desirable. For a similar reason it were probably better that a number of cases occurring during a succession of years should be subdivided—those occurring within the space of one or two successive years being grouped together, and each group analyzed separately. In view of this consideration, I have deemed it best to analyze the cases I have already collected, without waiting longer to increase the number.

Of the fifty two cases which have furnished the data for the examination upon which I wi l now enter, *fourteen* occcurred in private practice, and the remainder, *thirty-eight.* were observed at the *Buffalo Hospital of the Sisters of Charity.* I shall consider these as forming two distinct groups. The cases from private practice are recorded less fully than those at the Hospital. They run through a longer period of time, the first case being observed in December, 1845, and the last in June, 1850—a space of five years. The hospital cases were all observed between August, 1848, and March 1850—a space of eighteen months. The circumstances surrounding the patients in private practice, would, of course, be expected to differ in different cases. On the other hand, at the hospital, the patients were similarly situated. Inasmuch as the hygienic condition incident to hospitals, as well as private families, tend to modify, more or less, the symptoms and progress of a disease, frequently preventing a fair exemplification of its natural history, itl should be premised that the *Hospital of the Sisters of Charity* presents in all respects a most favorable combination of circumstances for the relief of the sick. The wards, during the time these cases occurred, were not crowded, and were extremely well ventilated. The attendance and nursing could hardly be improved, and was much better suited to the welfare of patients, than in the private houses even of those who can command all the comforts of life. The personal attentions of family and friends, in severe illness, are often unskillful and injudicious, and money cannot purchase the discreet and faithful offices of a *Sister of Charity!*

The records of the cases in private practice were not made at the time of the daily visits ; sometimes no record was made for several days, and, in a few instances, the details were noted from memory as the disease was about to terminate.

The histories of the cases in the hospital consists of daily records, (with occasional exceptions of a day or two,) made by myself at the bedside, at the diurnal visit, which was usually in the morning. I can therefore vouch that they are as accurate as I had the ability to make them. I must confess, however, that while arranging the facts for examination, I had occasion to regret that the records had not contained some particulars of which frequently no mention is made, either positive'y or negatively. In this respect the histories are far less complete then I could wish them to be. This, however, does not affect the accuracy of the analysis in so far as the facts are recorded.

The phrase *Continued Fever* is used, generally, in a generic sense, em_

bracing two or more species of febrile disease. The distinctions between what have been termed *typhus*, and *typhoid* fevers, have, of late years, occasioned much discussion by medical writers, and practitioners are not agreed whether to regard the different types thus distinguished, as really and essentially distinct forms of fever, or not, some contending that they are different febrile affections, others that they are essentially identical. The views which the writer has hitherto entertained on this point are, that cases of continued fever admit of being nosologically discriminated after this division, but that there do not exist sufficient grounds for regarding the two forms of disease as radically and essentially distinct; in other words, that continued fever is one disease, and that the divisions which it may be convenient to make, are based on differences which are nosological, rather than pathological, the uniformity of which, even for nosological purposes, not being perfectly reliable. The distinctions between Typhus and Typhoid fever, and the subject of diagnosis, will come up more appropriately hereafter. The facts contained in the cases under examination will be studied with reference to the points just mentioned, and, for this end, it becomes necessary to discriminate beforehand, between those cases which have claims to be considered as cases of *Typhus*, and those which are properly cases of *Typhoid* fever. Applying the rules of discrimination as laid down by those writers who maintain that the two forms of disease are essentially distinct, and rejecting as indeterminate all cases in which there exists any room for doubt as to the type, the result is as follows: of the *fourteen* cases in private practice *twelve* are distinctly cases of *Typhoid* fever; one was a case of *Typhus*, and one is *doubtful* from absence of sufficient data in the record. Of the *thirty-eight* hospital cases, *eighteen* were cases of *Typhoid*, and *twelve* were cases of *Typhus* fever; *eight* admitted of some doubt.

Another basis for a division of cases in order to institute comparison of the facts belonging in each, is the issue of the disease. Of the *fourteen* cases in private practice *four* were fatal. Of the *thirty-eight* in hospital practice *thirty-one* recovered.

Of the fatal cases in *private* practice, in *two* the disease was of the *Typhoid* type, in *one* the type was considered doubtful, and the only case of *Typhus* in this group proved fatal.

Of the fatal cases in hospital, *two* were of the *Typhus* type, *four* were of the *Typhoid* type, and in *one* the case was doubtful.

The ratio of mortality between the hospital cases, and those in private practice will be observed ; the former being about 13½, and the latter 22½ per cent., showing, in so far as a comparison of these two groups of cases go, a superiority in the hospital management of the disease.

Something should, perhaps, be said concerning the correctness of the diagnosis in all the cases set down as cases of continued fever. In places where both remittent and continued fevers prevail, practitioners sometimes confound the two, and unless the reader can have faith to believe that this, or any other error of diagnosis has been avoided in the present collection of cases, he can, of course, attach little or no importance to the results of the analysis. It is obviously difficult for the writer to furnish evidence of his competency to discriminate, practically, the disease under consideration, from other affections. The reader must estimate, as he best may, the grounds for reliability on this score. This assurance, however, may be given, that the utmost care has been taken to admit into the collection those cases only which, in the writer's opinion, were undoubtedly cases of continued fever, all recorded cases which seemed to him in the least degree doubtful, having been rejected.

In proceeding now to present the results which may be developed by the study of the cases under consideration, together with such considerations as, in connection therewith may be suggested, it will be most convenient to follow the order pursued in the analytical process already performed, adopting as many different sections as there are classes in which the events, symptoms, etc. of all the cases have been distributed.

### SECTION FIRST.

*Age, Sex, Occupation, Civil Condition, Nativity, Habits, Season, Constitution and previous health of the Patient, Period of residence in this Country and in this City, Duration of the disease before coming under observation.*

*Age.*—The ages of the private patients are given in all but two cases, i. e., in *twelve* cases. Of these *ten* are cases of *Typhoid* fever. The maximum age of these cases is *thirty-four*, the minimum age, *seventeen*. The mean age is 23 7-10. Of these *ten* cases, *two* proved fatal. The ages in these *two* cases were 21 and 34. The only case of Typhus in this group of private cases proved fatal, the age being 25. The only case indeterminate as to its being *typhoid* or *typhus*, proved fatal, the age being 40.

In the *hospital* cases, the ages are given in all but three cases. Of *seventeen* cases of the *typhoid* type in which the ages are given, in *one* case the age was 45; in *one* case the age was 35; in *one* case, 24. In the case of the youngest patient the age was 13. In the other cases, 13

in number, the patients were between 17 and 24 years of age. In six cases the age was precisely 20. The mean age is 22 2-17.

Of *ten* cases of *typhus* in which the ages are given, in one case the age was 50. In *four* cases the age was 20, which was the minimum. The mean age is 26½.

Analysis of large collections of cases of *typhoid* fever by Louis, Chomel, Jackson, and others, have established the existence of certain laws as respects age in this disease. The vast majority of cases occur between the ages of 15 and 30. Cases occurring after 40 are extremely rare, although they are occasionally met with. *Typhus* fever, on the other hand, evinces far less regard for age.

The results given above, correspond very closely with those obtained from observations by the authors just named, on a larger scale. In the collection of hospital cases analyzed by Dr. Jackson, of Boston, embracing *two hundred and ninety-one* cases, the average age was found to be about 22⅛ years, which, it will be perceived, is the same, with a slight fractional difference, as in the analysis of the hospital cases just made.

The cases of Typhus in our collection exhibit a higher maximum, and a greater mean. The latter, however, is less than it would seem to have been in the observations of others. Prof. Jenner, of the University College, London, in a late report on this subject, states that of forty-three fatal cases of typhus, nearly *one-third* were more than *fifty* years of age. —[*Amer. Journal of Med. Sciences, No. for July,* 1850.]

It remains to compare the fatal cases with those in which recovery took place, with respect to age. The observations of Louis go to prove that the average age of those who recover is less than of those who die with typ oid fever.

Of *sixteen* cases of *Typhoid* in which the ages are given, 4 proved fatal, and 12 recovered. Among the fatal cases was the case presenting the maximum of age (45), and also the case presenting the minimum age (13).

The mean age in the cases that proved fatal, is 24½. The mean age in the cases in which recovery took place is 21 8-12.

Of *eleven* cases of *Typhus,* in which the ages are given, *two* only proved fatal. Among the fatal cases was the case of the maximum age, 50, and the mean age, is 35. The mean age in the cases that recovered is 25 5-9.

The above comparison refers to the hospital cases.

In the group of *private* patients, of 8 cases of *Typhoid,* in which recovery took place, the mean age is 22 6-8. Of two fatal cases, the age in one was 21, and in the other, 34, giving a mean of 27½. In the only falal case of typhus the age was 25.

In so far as our cases afford results, then, they sustain the deduction of Louis, that the disease is less likely to prove fatal in proportion to the youth of patients.

*Sex.*—In the cases of Fever analyzed by Louis, and Jackson, by far the greater number were of the male sex. Some instances, however, are mentioned by Dr. Bartlett, in his work on Fever, of collections of a large number of cases in which the number of females preponderated.

In the cases which I have preserved, the number of males very much exceeds that of females.

Of the *fourteen private* patients, all but two were males. Of the *hospital* patients, the number of males affected distinctly with *typhoid* fever, was 17; of females, 1. The number of males affected with *typhus* fever was 10; of females 2. Of the cases in which the diagnosis was indeterminate, five were males and three females.

The disparity in the hospital cases might perhaps be accounted for by the fact that a larger number of males resort to such institutions than females. This explanation, however, will not apply to cases in private practice: and, hence, in so far as from the small number of this group of cases, any inductions are admissible, the inference is, that females are much less likely to be attacked with continued fever than males.

In the cases analyzed by Dr. Jackson, of Boston, the average age of females affected with fever was found to be somewhat higher than that of males. The number of female patients in my collection is too small for comparison on this point.

*Occupation.*—With reference to the *occupations* of the patients, no relations of the disease to any particular kinds of employment are developed. Of the patients treated in hospital, *three* were mariners, *six* were mechanics of different trades, *fifteen* were laborers, *one* was a *waiter*, one a clerk, *one* a porter, *one* a foreman in a steam mill, *one* a domestic, (female), *one* a chamber-maid, *one* a steamboat cook, and *two* were Sisters of Charity. Of the cases in private practice, *four* were soldiers recently enlisted, *two* were students, *one* a physician, *one* a maker of patterns for castings, *one* a laborer, *one* a housewife, *one* a female without occupation, *one* a male without any particular employment.

Analysis of much larger collections of cases have failed to show the existence of any tendency in particular vocations to favor the development of the disease. That the hospital patients were all in what are generally considered the humbler walks of life, will not be deemed singular, when it is considered that it is for the relief of this class that the institution is chiefly designed.

*Civil Condition.*—Of the *fourteen* patients in *private* practice, *one* only was married, a female 34 years of age.

Of the *thirty-eight hospital* patients, *one* only was married, *one* was a *widow* and one a *widower.* This, however, which might at first seem a striking result, proves little or nothing respecting any causative influence derived from the unmarried state, when it is considered that the average age of the patients is between twenty-two and twenty-six years, and also that, in so far as the hospital cases are concerned, comparatively few married persons, with any disease, enter the Institution.

*Nativity.*—Of the patients in *private* practice, *eight* were Americans, *three* were Germans, *one* was an Irishman, *one* an Englishman, and in one case the nativity is not stated.

Of the *Hospital* cases, the nativity of the patients with *typhoid,* is as follows: Americans, one; Irish, seven; Germans, ten. Of the patients with typhus as follows: Americans, one; Irish, eleven; Germans, none. Of the doubtful cases as regards the type of the fever, as follows: Americans, two; Irish, five.

I had doubted whether it were worth while to take the trouble to ascertain the nativities, but the result, in one point of view, is certainly curious. Of twelve patients affected with typhus, all but one were Irishmen. The reader is doubtless aware that Dr. Lombard, of Geneva, Switzerland, and others, have contended that true typhus is of Hibernian origin, and that its appearance in other countries is owing to its importation from Ireland. The result of the present analysis is not given as sustaining this idea, for the recency of emigration is obviously involved, and with respect to this point, the facts have not yet been examined; but the singularity of the coincidence, if it be nothing more, is worthy of note.

*Habits.*—In the *hospital* cases, the records, with very few exceptions, are defective in information respecting the *habits* of the patients.

In *eight* of the cases in private practice, the habits were good. In four the habits were unknown. In one the attack had been preceded by dissipation, and in one the patient was not intemperate, but irregular and licentious.

*Season.*—Observations have appeared to show that while the *typhus* type of continued fever is unaffected by season, i. e., liable to prevail equally at any portion of the year, the *typhoid* type, on the other hand, manifests a predilection for the autumnal months, although it is by no means restricted in its occurrence to the latter. In New England, where typhoid fever is the predominant febrile disease, it is commonly termed the *autumnal* or *fall* fever. The dates of the cases under analysis, were as follows:

*Private Cases—Typhoid.*—1845, December, *one* case; 1846, January, *one* case, October, *two* cases; 1847, September *two* cases, October, *one* case; 1848, August, *two* cases, September, *one* case; 1849, September, *one* case; 1850, June, *one* case. *Typhus*—1847, November, *one* case. Thus *eight* out of twelve cases of typhoid fever occurred during the months of *September, October, November* and *December.*

*Hospital Cases—Typhus.*—1848, August, *one* case, November, *one* case; 1849, March, *two* cases, June, *three* cases, July, *one* case, August, *one* case, October, *one* case; 1850, January *one* case, February *one* case, making 12. *Typhoid*—1848, October, *one* case, November, *one* case December, *one* case; 1849, March, *one* case, May, *one* case, June, *one* case, July, *one* case, August, *two* cases, September, *five* cases, October, *one* case, November, *two* cases, December, *one* case, making 18.

Thus, *nine* out of *eighteen* cases of *typhoid* occurred during the months of *September, October, November,* and *December, five* occurring in the month of September; while of the twelve cases of *typhus,* only two occurred during these months, one in October and one in November.

After the month of December, 1849, up to the time when my period of service at the hospital ceased, which was April 1st, 1850, not a single case of typhoid fever was received. During this period, two cases of typhus are recorded, and two cases in which the type is left indeterminate.

The result of the analysis, therefore, goes to sustain the correctness of the observations, above mentioned, respecting the relations of the two types of continued fever to the seasons.

*Constitution and health at the time of attack.*—In every one of the cases in *private practice,* the *constitution* of the patient might be pronounced good, i. e., none were suffering under chronic disease, or general ill-health. The same is believed to be true of the *hospital cases.* In many cases it is so stated in the records, but in the cases in which this statement does not appear, it is a fair inference that, were it otherwise, the fact would be recorded. In no case is it recorded that the patient possessed a feeble or broken constitution. In so far as my observations go, then, the conclusion is, that persons not possessing good constitutions, are not apt to be affected with continued fever.

*The state of health at the time of attack* is known in all the cases occurring in *private* practice. In all but two cases it was good. In one of the excepted cases the patient had a short time before had *Pertussis,* from which, however, he had recovered; in the other case, the patient was reduced by lactation, and was laboring under *stomatitis materna.* In the *hospital cases*, the health is stated to have been good in *seven* cases of *typhoid,* and four cases of *typhus.* It is not stated to have been poor in any case, and in nearly all of the cases in which no mention is made of this point, it may fairly be inferred that the health was good, from the patients having been arrested by the disease while pursuing their customary avocations, all of which would require for their performance good health, and from other circumstances of the history. Hence, it seems proper to conclude that, in so far as the present collection of observations affects this point, persons in good health are most apt to be attacked with continued fever.

By the *state of health at the time of attack* is meant, here, the condition prior to the first developement of the *prodromic* symptoms of the disease. The latter, which by some are considered to constitute a stage of the disease, and by others regarded as preliminary to it, will form a subject of inquiry under another division.

*Period of residence in this country, and in this city.*—The period of residence in the country, and town, where cases of continued fever are observed, has important bearing upon several questions relating to the etiology of the disease. If a patient have recently arrived from a foreign country, it becomes a subject of inquiry in how far may the production of the disease be due to circumstances affecting him before his arrival. Among the circumstances which may be suspected of being involved in the origin of the disease in these cases, are endemic and epidemic influences to which he may have been exposed prior to his embarkation, the confinement and hardships of the voyage, contagion, moral influence of change of country, etc.

*Cases in Private Practice.*—One of the cases was observed in a neighboring place, and is omitted. Of the remaining *thirteen* cases, *eight* had resided in this city for a period less than six months. Of the other *five* cases, *one* had resided in the city about a year, *one* about two years, and the remaining *three* had resided for a longer period. In *four* cases the patients had recently emigrated to this country. Among the latter is included the single case of *Typhus* in the group of private cases.

*Hospital Cases.*—Of the *Typhoid* cases the period of residence is recorded in *ten* cases, and nothing on this point stated in *eight* cases. In *seven* of the *ten* cases the patients had recently emigrated to this country. In *nine* of the *ten* cases, the patients had recently come to reside in this city. The periods in the cases respectively, as recorded, are as follows:—six weeks in the country, and four weeks in the city; ten days in the country, and three days in the city; thirteen days in the country, and ten in the city; eight weeks in the city; a few months in the city; five weeks in the city; three and a half months in the country, and eight days in the city; recently in the country; a year in the country, and four months in the city.

In the single case in which the patient had not recently come to reside in the city, the patient had been in the country fifteen months, and in the city twelve months. This was the longest period of residence in any of the *Typhoid* cases in which the records contain information on this point.

Of the *Typhus* cases, the period of residence is stated in *ten* cases, and the histories are defective on this point in *two* cases. Of these *ten*, the patients had recently arrived in this country and place, in *eight* cases. Of the two remaining cases, the patient in one case was a native, and permanent resident; in the other case, the patient had been a resident of the city for ten years. The periods in the *eight* cases respectively, as recorded, are as follows;—in the country six months; five or six weeks in the country; in the country a month; just arrived in the country; in the country a week; recently in the country, and six weeks in the city; twelve days in the country, and four days in the city; just arrived in the country and city.

*Duration of the disease before coming under observation.*—In *nine* of the cases in private practice, the disease was observed from its commencement. Of the remaining (5) cases, *one* came under observation on the *fourth* day after the attack, *two* on the *seventh* day, *one* on the *fifth* day, and *one* on the *tenth* day. Of the fatal cases, *two* came under observation at the commencement of the disease, *one* on the *seventh* day, and *one* on the *fifth* day. Of the *Hospital* cases, four are deficient in information on this point. Of the remaining thirty-four cases, five only came under observation at the commencement of the disease; and by the commencement of the disease I mean not to include the prodromic symptoms, which, of course, nearly always exist for a greater or less period before application is made for medical aid, or the relief of an hospital. The duration of the disease prior to observation, varied from *two* to *twelve* days; in the greater number of cases, the period was between *four* and *eight* days. Before coming to the hospital, some had received no treatment, others had been attended by regular Practitioners, and some by Empirics.

The only point of interest under this head, which occurs to me, is to ascertain if there seems to exist any connection between the duration of the disease before coming to the hospital, and the fatal result. Of the *two* fatal cases of *Typhus*, *one* came under observation on the *fifth* day, and *one* on the *fourth* day. Of the *four* fatal cases of *Typhoid*, *one* came under observation on the *eighth* day, *one* on the *eleventh* day, *one* on the *fifth* day, and *one* on the *first* day. Of the cases of doubtful type, the only fatal case came under observation on the *eighth* day. The mean duration in these cases is about *six* days, which is about the average duration in the cases which did not prove fatal.

### SECTION SECOND.

*The Access, its duration and symptoms. Circumstances supposed to have been concerned in the production of the disease.*

By the term access I mean to designate that period during which the disease appears to be forming, commencing with the first symptoms of disorder, and ending when the disease becomes fully developed. By some this period is regarded as a *stage* of the disease, sometimes called the forming stage ; others regard it simply as presenting precursors, preceding and ushering in the disease, or the prodromic symptoms of the disease, but not intrinsically belonging to it. Without stopping to present arguments for either of these views, I will proceed to examine the cases under analysis with reference to this period. The chief points of inquiry which possess interest and importance, relate to the *duration* of the access, and to its *symptomatology*.

*Duration.*—In the *twelve* cases of *Typhoid* fever occurring in *private* practice, the records are deficient in information respecting the *access* in *two* cases. In the remaining *ten* cases, without an exception, the period of access embraced several days; but in most of the cases the precise period is not stated. The record in *six* cases, simply states that it extended over several days; in *one* case, it is stated *four* or *five* days; in *one*, *two* or *three* days; in *one*, *three* or *four* days ; and in *one* the patient had been in ill health for three weeks.

In the single case of *Typhus* in this group, the period of access was *two* days.

The indefiniteness of these records denotes a feature of the access of the disease which writers on the subject have remarked, viz., the difficulty of fixing, with precision, the precise date of its commencement. Patients when asked respecting the time when they first began to feel ill, generally reply "several days," "three or four days," etc., apparently not being able themselves to decide on the particular day when they ceased to feel well. In such instances the symptoms come on gradually and imperceptibly. On this account it is not practicable always to determine the exact duration of the access.

Nor is it an easy matter to decide on the termination of the *access*. What denotes the commencement (or the second stage) of the disease? This question is not readily answered. The symptoms of the access are generally continued, increased in degree, but not changed in character; nor are any new characters developed which may serve to indicate, with certainty, the termination of the access. My rule has been to pronounce the access passed, and the fever established, when the patient is compelled to take to his bed, i. e., when he feels no longer able to sit up. This rule is somewhat arbitrary, and is by no means perfectly accurate. Some patients, from choice or necessity, will keep about much longer than others. Much will depend on differences in mental disposition as respects apprehension, prudence, etc., with regard to this point. But, with these objections, I know of no single event, or group of symptoms, more available, and in the majority of cases, more correct than this.

In *hospital* cases it is much more difficult to ascertain the duration of the access than in private practice, patients frequently being received after the disease is more or less advanced, when they are unable to give a correct account of its origin and progress. On examining the histories I have collected, I find them very deficient in information on this subject. Of the *eighteen* cases of *Typhoid*, the information is very indefinite in *twelve* cases; in *four* cases, the disease appeared to have come on with a sudden attack. Of the remaining *two* cases, in *one* the access was of several days' duration, and in the other, *four* days. It is to be presumed that in all instances in which it could be ascertained that the disease came on suddenly, the fact was recorded; and that when the records are silent on this point, the disease came on, as is usual, with an access of several days' duration.

Of the *twelve* cases of *Typhus*, the records are defective in *five* cases; the attack was sudden in *one* case, the access was "several days" in duration in *four* cases, *five* days in *one* case, and *seven* days in *one* case. Of the *eight* cases of doubtful tpye, the attack was sudden in *one* case; the access was "several days" in duration in *three* cases, two or three days in

*one* case, and in *one* case the patient had been ill three weeks, (symptoms not ascertained,) and had recovered sufficiently to go to work, when he was suddenly attacked. In *two* cases, the records contain no information.

Of the *three Typhoid* cases proving fatal, the disease came on suddenly in *one*, and the duration of the access was not ascertained in the remaining *two* cases.

Of the fatal cases of *Typhus*, the attack was sudden in *one*, and the duration of the access unknown in the other case.

In the fatal case among those of doubtful type, the access was of several days duration.

The only conclusion to be drawn from the foregoing rather unsatisfactory analysis, is, that generally the access extends over several days, but that the attack, in a small proportion of cases is sudden—a conclusion according with general experience.

The data do not warrant a comparison of the two types in order to ascertain in which is the disease most likely to commence suddenly, and in which the average duration of the access is longest ; nor is the comparison of the fatal and not fatal cases, in these respects, of any value.

*Symptoms of the Access.*—The records of the cases are far from being comprehensive as respects the symptomatology of the access. This is, in part, because sufficient pains were not taken in noting the symptoms, and in part, because in a large proportion of cases, (more especially those in hospital) it is difficult, and, indeed, in many instances impossible to obtain a full account of the history prior to the patients coming under observation. In the cases in which the history embraces some mention of the symptoms of the access, generally the circumstances only which are striking, or occasional in their occurrence, are noted, those being omitted which are constant or quite common. I do not, therefore, deem it worth while to count the number of times each particular symptom is mentioned, with a view to determine the relative ratio of its occurrence, but I will simply give a list of the symptoms, with brief comments. They are as follows :

*Cephalalgia*, a frequent symptom in the cases of both Typhoid and Typhus ; *anorexia*, an almost constant symptom ; *chills*, in a few cases, with *rigors* more rarely, oftener *chilly sensations or slight shivering; pain in limbs ; pain in loins*, not so frequent, or prominent symptom as the last ; *sense of debility ; lassitude; cough* in three cases of Typhoid, in no case of Typhus, complicated with soreness in the chest in *one* case, and with pains beneath the sternum in one case ; *Diarrhœa*, in one case of Typhus, (a fatal case,)

and in four cases of Typhoid ; *nausea*, and in *three* cases *vomiting*. Of the three *fatal* cases of Typhoid, the following are the symptoms of the access noted, which are more or less peculiar to these cases ; in *one* case *delirium at night ;* and in one case *tremulousness in the muscles of the face* as in mental agitation, in *one* case *vomiting* and *diarrhœa*.

Of. the the three fatal cases of Typhus, in two the symptoms of the access were not noted, and in the remaining case the symptoms were, *cough' headach, pains beneath sternum, anorexia, moderate thirst, vomiting and diarrhœa*.

*Circumstances supposed to have been concerned in the production of the disease.*

In obtaining the previous history of cases coming under observation pains were not taken to inquire concerning the circumstances which might seem to stand to the disease in some causative relation. In the majority of cases nothing is recorded on this subject. Occasionally, however, the patients themselves mentioned circumstances to which they attributed their illness. The instances of this kind, and the supposed causes, and the type of the disease, are as follows :

Excesses of dissipation, continued for several weeks, to which the patient had previously been unaccustomed. (Typhoid.)

A catarrh, or mild bronchitis, accompanied by cough and soreness in chest, in two cases. (Typhoid.)

Baithing in cold water. (Typhoid.)

Exposure to cold and wet on the day prior to the attack, the patient becoming much chilled. (Typhus.)

Drinking cold water when heated. (Typhoid.)

Falling into the canal, and working all day in wet clothing, the patient being obliged to take to his bed on the following day. (Typhoid.)

Several of the patients thought their illness came from "taking cold," but without their having had any symptoms which constitute what is called a "cold," and the attack not succeeded any notable exposure. Many evidently referred their illness to cold under the common impression that most diseases thus originate.

Of those who had recently arrived in this country, the inquiry was generally made, whether sickness prevailed on ship-board ; in two instances only was it ascertained that this was the fact. Sometimes the answers to the inquiry were unsatisfactory, but in several instances the patients were certain that no febrile disease existed during the passage.

In the great majority of the cases, no cause for the disease is assigned.

In the few instances given in which causes were specified, it will not, of course, be supposed, from their being embraced in their histories, that much importance is attached to them. It is not probable that the circumstances mentioned by the patients as causes, really were so, or, at most, they are only to be regarded, as possibly, exciting causes. And so far as my observations go, continued fever frequently, if not generally, occurs without being preceded by any events which, even in the estimation of patients, stand to the dieease in the relation of cause; and, hence, it is a fair presumption in those instances in which an attack follows any of the common causes of disease, that their agency, if they have any, is of a subordinate kind.

In three cases facts connected with the development of the disease seem to have a bearing upon the question of *contagion.* One of the cases referred to, occurred in private practice. Twenty days before the development of the disease, a brother of the patient arrived in the city on a canal boat, and was transferred, first to a hotel, and, two days afterward, to the hospital. He was attended by me both at the hotel and hospital, but a record of the case was not made. The character of his disease was not determined until after his removal to the hospital, when it proved to be a case of Typhoid fever, with the characteristic eruption, etc. While at the hotel the two brothers occupied together a small bed room, and the then well brother, not only remained during the night, but officiated as an attendant throughout the day. The type o the disease, developed twenty days afterward, was typhoid ; the case was very mild, and unusually short in its duration, terminating, with a critical perspiration, on the fifth day. A few rose spots were observed on the chest and abdomen. In view of the circumstances just mentioned, it seemed fair to *suspect* that the disease was communicated from one brother to the other.

In the other two cases in which a contagious principle seemed to be involved, the patients were *Sisters of charity* at the hospital. To the two *Sisters* who had fever, had been assgned the care of all the fever cases, (with the exception of three cases) during the preceding winter, up to the time of their illness. They, alone of all the inmates of the house, became affected with fever. The first that became affected with fever, (sister H.) was attacked in February. She was attacked early in lent, and had conformed to the rules of abstinence prescribed by the Catholic Church for this season, continuing her laborious duties as before. This is mentioned as a circumstance to which perhaps, something is due in accounting for the development of the disease at that time. The disease was mild, attended with some delirium, no abdominal symptoms, without an eruption, terminating in convalescence on the *ninth* day. The case is included among those of *doubtful type.*

The second (sister N.) was attacked about a month afterward, in March, 1850. Her duties, in consequence of the illness of sister H., had been for the previous four weeks much increased. This case was milder than the preceding, and shorter in duration. It was unattended by abdominal symptoms, and an eruption. It is also included among the cases of *doubtful type.*

That thus the two persons who were especially brought into close and continued proximity to the fever patients, should have contracted fever, no other case of fever being generated within the Institution, certainly can be less rationally explained by the law of probabilities, than by the supposition of contagion.

Assuming that these instances do exemplify the operation of a contagious principle, the facts accord with the views now generally entertained respecting the communicability of Continued Fever, viz., that the disease may be diffused by contagion, which differs in intensity at different times and places, but that generally the disease is not developed except after long continued exposure to the contagious miasm, or under circumstances in which the miasm is greatly concentrated, and, even then, co operating causes seem often to be required to determine the attack.

It is supposed by those who recognize *Typhoid* and *Typhus* as distinct types of Continued Fever, that the latter is either exclusively, or much more liable to be communicated than the former. The first of the instances just stated, assuming the supposition of contagion to be correct, would establish the communicability of Typhoid. The two latter instances cannot be considered to have any bearing on this point, inasmuch as the patients had been brought into contact with both types of the disease.

(*To be continued.*)

