# Errata in the Report on Continued Fever, by the Editor

**Published:** 1850-11

**Authors:** 


*Errata in the Report on Continued Fever, by the Editor.*—In stating the ratio of mortality in the hospital and private cases of Continued Fever, in the Report by the Editor of this Journal, page 136, No. for August, 1850, a material error in figures inadvertently escaped notice, which the reader is requested to correct. For the numbers 13½ and 22½ per cent., read 18 8-19 and 28 4-7 per cent.

